# First person – Mai Nguyen

**DOI:** 10.1242/bio.059519

**Published:** 2022-07-27

## Abstract

First Person is a series of interviews with the first authors of a selection of papers published in Biology Open, helping early-career researchers promote themselves alongside their papers. Mai Nguyen is first author on ‘ Cytoneme-like protrusion formation induced by LAR is promoted by receptor dimerization’, published in BiO. Mai conducted the research described in this article while a PhD student in Makoto Sato's lab at Osaka University, Japan. She is now a researcher] at Meiji Co., Ltd., Tokyo, investigating the formation of membrane protrusions in cellular communication.


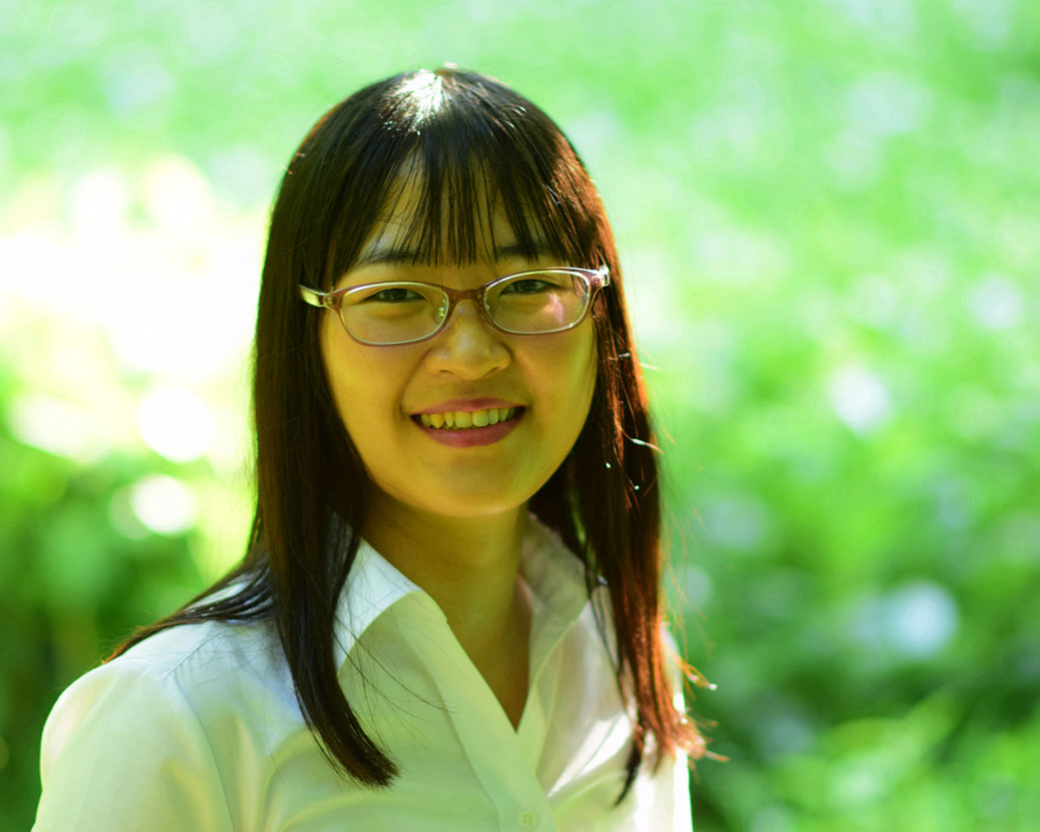


**Mai Nguyen**

**Describe your scientific journey and your current research focus**

I was fortunate enough to be funded by the Japanese Ministry of Education, Culture, Sports, Science and Technology (MEXT) throughout my studies from undergraduate to PhD, which gave me the opportunity to build up foundation in biological sciences and further my education in molecular biology in the field of neuroscience. During my undergraduate degree, I had a chance to do research in the Laboratory of Synaptic Plasticity (Dr Akihiko Ogura's Lab) at the School of Science, Osaka University, where I performed research on the effects of excess stress on memory formation in the developing brain. Then I became interested in how such brain functions are implemented, directing my attention to how neural circuits are formed. Eager to study more about neural circuit formation, I joined the Department of Anatomy and Neuroscience (Dr. Makoto Sato's Lab) for a 5-year doctoral program at the Graduate School of Frontier Biosciences, Osaka University. My work there focused on the molecular mechanism of circuit formation in nerve fibers that extend from the cerebral cortex to the subcortex, focusing on the corticospinal tract. To my surprise, I discovered the ability of LAR to induce long and branching protrusions in HEK293T cells, which led me to pursue this exciting research topic.

“To my surprise, I discovered the ability of LAR to induce long and branching protrusions in HEK293T cells”

**Who or what inspired you to become a scientist?**

As a child I was always eager to learn more and discover things on my own. I enjoyed doing simple experiments described in the encyclopedia that my family gave me as a gift. My inspiration for science, however, became clear when I was studying at primary school. I was very impressed with the images of the cells when I first saw them in a textbook titled Przyroda, meaning ‘Nature’. As I remember correctly, there were images of animal and plant cells together with the text explaining their structural differences. I was instantly fascinated by the fact that the cell is the smallest unit that constitutes the human body. From that very moment, I decided to become a biologist, and I have pursued the path of becoming a scientist ever since.

**How would you explain the main finding of your paper?**

Humans are multi-cellular organisms. In order for such organisms to function, cells need to communicate with each other. Until now, cellular communication has mostly been categorized into four major types: direct contact, paracrine, endocrine, and synaptic (neuronal). However, in addition to these well-characterized methods for cell signaling, an increasing number of studies have shown the importance of a special type of cellular protrusion called a cytoneme, meaning ‘thread-like cytoplasm’. In this research, we have shown that a transmembrane protein called LAR and its dimer formation stimulate the growth of ultra-long and complexly branched cytoneme-like protrusions in mammalian cells. Our results emphasize the importance of LAR function in all of the processes of protrusion formation: from initial growth to elongation, and, importantly, branching, which supports cellular communication.

**Figure:**
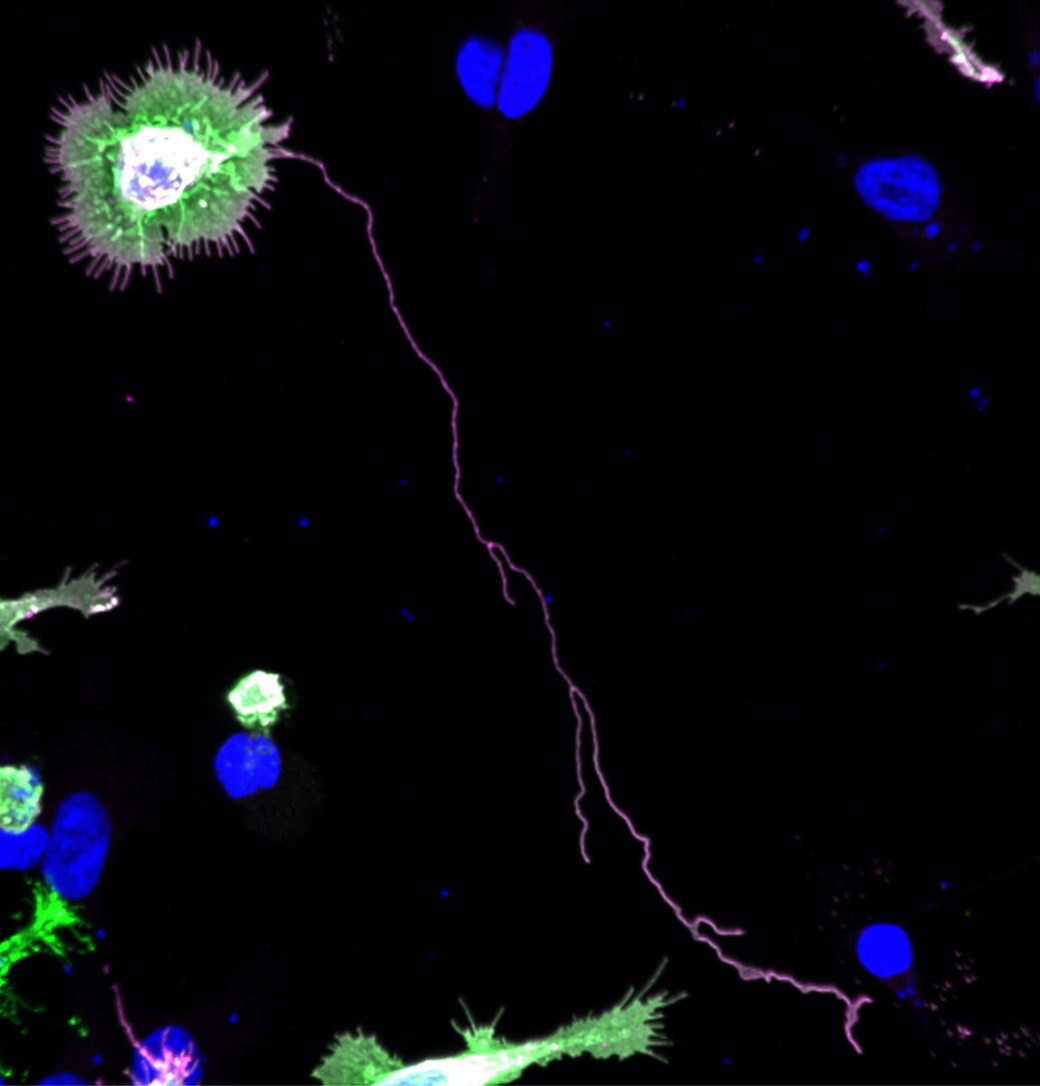
**The formation of long and branching cytoneme-like protrusions is promoted by leukocyte common antigen-related (LAR) receptor protein tyrosine phosphatase dimerization.** This merged confocal image shows a HEK293T cell expressing dimerization-inducible pair of LARs: V5_LAR_FRBLgBiT (green) and Myc_LAR_FKBPSmBiT (magenta), in which formation of an ultra-long and branching protrusion was observed following 24 hours of rapamycin application for LAR dimerization induction.

**What are the potential implications of this finding for your field of research?**

Although previous studies have shown the importance of thin membrane protrusions, including cytonemes, in cell signaling, a complete understanding of the mechanism by which these structures are formed is lacking, especially in mammals. Our findings, thus, contribute to the understanding of how these morphological features are established, and suggest their possible role in the mammalian cellular system. I believe our findings might be the foundation of describing a common mechanism by which membrane protrusions are formed, facilitating cellular communication.

“I believe our findings might be the foundation of describing a common mechanism by which membrane protrusions are formed, facilitating cellular communication.”

**Which part of this research project was the most rewarding?**

Observing the intriguing phenomenon of non-neuronal cells forming ultra-long and complexly branched protrusions was fascinating. The fact that this finding was unexpected made the experience even more rewarding!

**What piece of advice would you give to the next generation of researchers?**

If you find something that interests you, follow it. It may feel difficult in the moment, especially when you don't know the outcome and there are more questions than answers. However, do not give up just because others disagree with your idea. Instead, cherish it and test it empirically. I think it is crucial to focus on the excitement that your research brings and its future implications. These will give you motivation to continue your scientific journey, which I believe is the key to great discoveries.

**What's next for you?**

After graduating from the PhD program, I began my research career at Meiji Co., Ltd. I look forward to broadening my research interests and collaborating with researchers from other fields of studies. I hope this will allow me to make many remarkable discoveries in the future!
